# Experiences of Using a Continuous Glucose Monitoring System in Children—A Descriptive Study with Parents in the Republic of Georgia

**DOI:** 10.3390/healthcare9111556

**Published:** 2021-11-16

**Authors:** Nino Kheladze, Lars Kristensson, Anders Johansson, Elizabeth Crang-Svalenius, Bodil Ivarsson

**Affiliations:** 1 M.Iashvili Children’s Central Hospital, Evex Medical Corporation, Lubliana Street 2/6, Tiblisi 0159, Georgia; 2 Office of Medical Services, University Trust, Region Skåne, SE-221 85 Lund, Sweden; lars.kristensson@skane.se (L.K.); anders.johansson@med.lu.se (A.J.); bodil.ivarsson@med.lu.se (B.I.); 3 Clinical Sciences, Lund, Faculty of Medicine, Lund University, SE-221 85 Lund, Sweden; 4 Health Sciences, Faculty of Medicine, Lund University, SE-223 40 Lund, Sweden; elizabeth.crang_svalenius@med.lu.se

**Keywords:** CGM units, children, developing country, diabetes disease, families, FGM units, psychosocial aspects

## Abstract

The benefits of medical devices are often multifaceted and may have an important impact on patients’ and relatives’ physical, mental and/or social well-being. Diabetes is a metabolic disorder and a continuous subcutaneous glucose monitoring sensor can suggest increasing treatment satisfaction. The purpose of this study was to describe parents’ experiences during their daily lives and support needs when a child uses a Flash Glucose Monitoring system (FGM). Twenty parents (*n* = 3 men vs. *n* = 17 women) to children (age ranged between 22 months and 16 years) with diabetes disease type 1, treated with an FGM unit (used for an average of 7 months (range 1–72)) at home, participated in this study. A qualitative questionnaire survey with open questions including follow-up dialogues was distributed to the parents, and collected data were analysed using qualitative content analysis. *Overall satisfaction* with the Libre device was Md 10 (IQR 9.25–10). One main theme “Advances in technology significantly improved everyday life” emerged from 2 categories: Improvements in quality of life and Elements of challenges. In conclusion, this qualitative study determined that parents of children with DMT1 experience a great improvement in daily life when given the opportunity to use the Libre device.

## 1. Background

More than 1.1 million children and adolescents under the age of 20 live with type 1 diabetes [1]. Diabetes is characterized by complete or partial lack of insulin, which leads to elevated blood sugar (glucose) levels and the overall prevalence of diabetes has been estimated to be about 8% of a population [2]. The insulin deficiency requires lifelong insulin therapy and type-1 diabetes (T1DM), accounts for 5–10% of the total cases of diabetes worldwide. The annual increase of childhood diabetes is estimated at around 3%. The cause of the disease is not known, but both heredity and environmental factors are considered able to contribute [3].

Good control of the blood glucose level is important to avoid the sequelae of diabetes. Blood glucose levels are affected by several factors such as diabetes medications, dietary intake, and physical activity and needs to be evaluated both in the short and long term. Blood glucose can be measured by the patient themselves with test sticks (self-monitoring of blood glucose, SMBG) or continuously via a continuous subcutaneous glucose monitoring (CGM) sensor. If CGM is not available, 7 to 10 plasma glucose checks per day are usually needed for satisfactory glucose control. This is quite stressful and painful for most children especially young children and very disturbing at night [4].

Since blood glucose determinations only provide the present level, there was a need for continuous glucose measurement and in the year 1999 the first apparatus for this purpose was introduced on the market [5]. In recent years, and available in Europe since 2014, the FreeStyle Libre Flash Glucose Self-Monitoring System (FGM-method using a sensor for monitoring interstitial fluid glucose) was introduced to help individuals with type 1 and 2 diabetes, to achieve better glucose control [6].

Diabetes is a long-term condition that can have a major impact on the life of a child or young person, as well as on their family or caregivers. In addition to insulin therapy, diabetes management should include education, support, and access to psychological services. A European multicenter trial, including 70 parents from United Kingdom, Ireland and Germany, showed improved treatment satisfaction regarding CGM use [6]. A review found similar results, but also that the clinical effectiveness of the FGM system needs to be further investigated [7]. Future trials should therefore include quality of life outcome assessments, at various points, to improve clinical and cost-effectiveness modelling.

In the Republic of Georgia in the South Caucasus, the government provides universal access to health care. Regarding diabetes management of children, this is government-funded, but the inhabitants must still pay for some healthcare directly out of pocket [8] and the cost of FGM is a part that the family must bear.

A family-centred perspective, where healthcare professionals collaborate with the whole family in all aspects of care [9], allows the opportunity to evaluate medical product characteristics as a part of healthcare.

Since these types of investigations from developing countries are rare, this study aimed to elucidate Georgian parents’ experiences of their daily lives when a child in their family uses an FGM device.

## 2. Materials and Method

### 2.1. Design and Informants

This study used a qualitative, descriptive approach following SRQR statement for reporting quantitative research [10]. A purposive sampling methodology (20 informants) was used to recruit informants (parents) of children with T1DM [11]. Initially, nine (*n* = 9) informants were recruited from a parent group on social media, and then eleven (*n* = 11) informants were selected from an outpatient clinic. In line with the country’s ethical guidelines, approval was obtained from the M. Iashvili Children’s Central Hospital, Tbilisi, Georgia (Dnr: Hospitals-OUT-21-0517-2890). The investigation conforms to the principles outlined in the Declaration of Helsinki [12]. The informants were guaranteed confidentiality and informed that they could withdraw from the study at any time.

### 2.2. Data Collection

The questionnaire survey focused on the parents’ experiences of care when a child in their family uses an FGM device. The questions, inspired by the MedTech20 questionnaire [13], and experiences of the first author (paediatric endocrinologist), were collaboratively created by the study team (Appendix A). The MedTech20 questionnaire is a generic questionnaire and offers the possibility of elucidating the patient-perceived experience for different medical devices [13]. The questions were then tested to check their relevance and clarity.

In an introductory letter, all the informants were given written information about the study, its aim, and the questionnaire survey including the follow-up dialogue. The informants answered the questions and sent them back to the first author (NK); thereafter the informants were contacted by ‘phone (NK) for follow-up questions and additions. The complemented follow-up questions were: “Can you tell me more?” and “What did you feel/mean?”, for clarification and to continue the conversation. The present study includes all parents after informed consent, and data collection was started during the second quarter of the year 2021. Inclusion criteria of the informants were parents of children with T1DM and treated with an FGM unit at home.

### 2.3. Analysis

**Figure 1:**
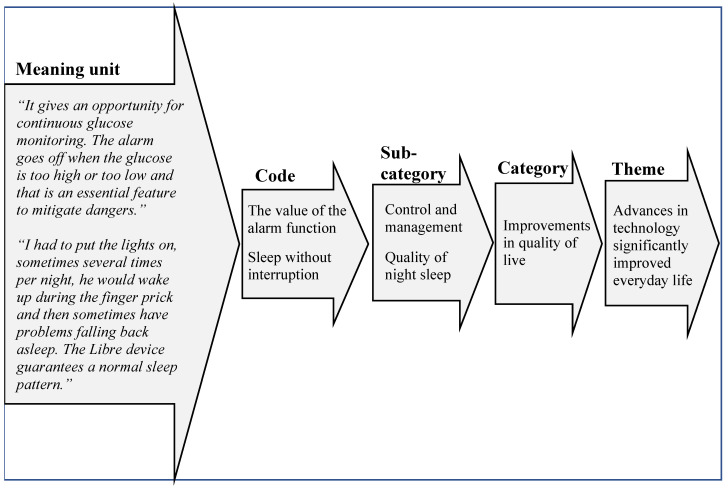
Example of the analysis process, from meaning unit to main theme.

The questionnaires completed by the parents were written in the Georgian language and then discussed with author NK by telephone. Thereafter they were transcribed and translated verbatim to English by author NK (native Georgian with one year scholarship in USA). The transcript was analysed using qualitative content analysis as described by Graneheim & Lundman [14] and was read carefully several times by all authors (one native English) to create a good overview of the material. Two authors, EC-S and BI, identified codes that were sorted into subcategories and categories. Through the final process of analysis, there were repeated discussions about the subcategories and categories between all authors until an agreement was reached. An example of the analysis process is shown in Figure 1. Quotes from the interviews were used to underpin and reinforce the results.

Collected demographic values were analysed with the statistical software SPSS^®^ version 27.0 (SPSS Inc., Chicago, IL, USA). Parents’ and children’s demographics are presented as absolute- and relative frequencies and the age of the children was clustered in the following groups: 0–3, 4–7, 8–12, and 13–16 years. An Overall satisfaction with the Libre device is described with a median (Md) and interquartile range (IQR) value, graded on a 10-point Likert scale (1 = Not at all satisfied; 10 = Completely satisfied).

## 3. Results

### 3.1. Descriptive Findings

Table 1 presents the parents’ and children’s demographics and the children’s length of diabetes duration, along with the parents’ Libre device experience in months. The study included 20 parents (*n* = 3 men (15%) vs. *n* = 17 women (85%)), with a mean age of 38 years (SD ± 4) (Table 1). The respondent’s Overall satisfaction with the Libre device was Md 10 (IQR 9.25-10).

### 3.2. Qualitative Findings

**Figure 2:**
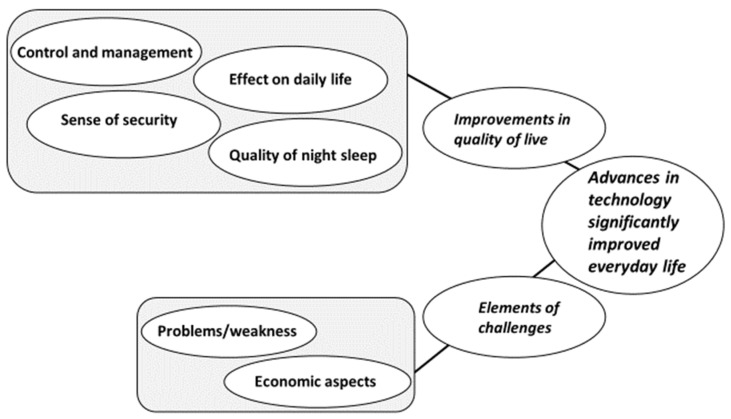
Subcategories, categories, and a main theme.

The content analysis emerged into one main theme; “Advances in technology significantly improved everyday life”, from two categories; Improvements in quality of life & Elements of challenges, based on six subcategories (Figure 2).

#### 3.2.1. Improvements in Quality of Life 

##### Control and Management

The device improved the quality of life for both parents and children. Most parents were also relieved not to have to take blood samples continually. Parents felt it was crucial for the management of diabetes and also gave them a better understanding of the illness. In some cases, the child had not had the device for long enough to have obtained HbAlc levels. These parents were, however, satisfied with the continuous readings. All parents agreed that they found it easier to adjust insulin doses and that the children’s glucose levels were much more stable. Most families used the Libre II device, which has an alarm function for high or low blood glucose levels. Using this function, the parents describe an even better management of glucose levels, as this provides continuous reliable data and shows trends.

“It’s a huge comfort for us and our child is so relieved. Her fingertips even become softer. I am now able to understand the condition better, things that I didn’t understand before, and realise they are becoming much clearer.”

(R13)

The daily graphical data was also appreciated and gave better knowledge of the disease. The general opinion was that the device was very easy to use and trend data were useful for contact with healthcare providers and for the prescription/dosage of insulin. A few parents had been given support at the start from another parent already acquainted with the device. 

Some friends and acquaintances were inquisitive about the FGM and many of the children enjoyed explaining what its function was. 

The older children could take a more active part in the management of their diabetes. For the parents, it was invaluable and essential. It enabled them to monitor the child’s glucose levels from a distance, e.g., when the child was at school or nursery. This meant that the parent could take responsibility and did not have to rely on staff, who could have little knowledge of diabetes. This benefit of the device was of great assistance in decision-making.

“Very important. I was afraid to let her go to school without it. I was thinking I had to quit work, so I could be close to her school all the time before we got to use the Libre. She feels kind of cool with a fancy device.”

(R10)

##### Effect on Daily Life

All parents said they felt safer with the device and trusted it. They found it easier to predict hyper-/hypo-glycemia and, in so doing, decrease the number of unpredictable incidents. One parent felt safe because her child could see immediately the effect of eating sweets or something unsuitable, and in this way better understand the effect and make better choices.

“Of course. Before the Libre she would not consider what she was eating and she would not restrict sweets. Now, when she can see what happens with her blood glucose when she eats something unsuitable. I think it helps her with her food choices and overall understanding of her condition”.

(R9)

The device was a great help in decision making, especially when the children were doing intensive physical activity such as dancing or playing other physical sports. It made it much easier to adjust the dose and supply carbohydrates depending on the trends from the device.

“We have info about the trends and if it tends to go up or down. It’s super useful especially when my son plays rugby”.

(R6)

The older children were able to control their diabetes better and could adjust their activities or take something to increase their blood glucose, and the Libre device, therefore, meant more freedom. The device made other family activities possible and most of the children could manage to balance their glucose levels themselves before more active sessions. However, due to the pandemic, some parents and their children had not been able to take part in collective activities as yet.

“Due to the pandemic there is no school, so I can only imagine it will be a great help”.

(R4)

Some stigma was experienced attached to diabetes, but in general, the Libre device was considered ‘cool’ and the children were perceived to have a high level of acceptance. If the children for some reason did not want to expose the device, it was perceived as easy to hide.

“Most people don’t know what it is, my kid loves to explain to them”.

(R7)

##### Sense of Security

The Libre device gave the parents an overall sense of security. The parents mentioned that even the school staff felt more comfortable if the child had the device and some parents mentioned that it was a prerequisite for them to consider sending their child to day-care.

“It’s absolutely a must-have item. We can only let her attend day-care with this device”.

(R12)

However, some children/parents had only recently started using the Libre device and had no experience of acute- (hyper-/hypo-glycemia) and/or stressful situations. Nevertheless, the parents who had experienced such situations found the Libre device very helpful in controlling different situations, and especially mentioned were virus infections. The device, therefore, helped to reduce parental anxiety and was also perceived as important for the parents’ coping strategies.

“When our child had an acute viral infection, he had high blood glucose levels and the Libre device was essential for us to determine the insulin dosage”.

(R1)

Mostly, parents described the great reliability of the device, though only a few parents had initial difficulties in its introduction.

“The first time I had some help from someone who had experience, after that, I was able to do it easily”.

(R13)

##### Quality of Night Sleep

One great advantage of the device was at night-time. The Libre device had improved sleep quality for both parents and children. The advantage of not having to wake up the child during the night for traditional measurements was considered extremely valuable. For those who had the Libre II device, with an alarm function for critical glucose levels, this was experienced as even further improvement of the parent’s sleep during night hours. They could now relax without having to set an alarm clock to check the child’s blood glucose levels.

“We sleep much, much better. We wake up if the alarm goes off to take appropriate measures”.

(R1)

#### 3.2.2. Elements of Challenges

##### Problems/Weakness

According to the parents, very few of the children experienced any discomfort using the device. A small number had slight skin irritation, but one specific problem mentioned by many parents was keeping the device in place when doing physically energetic activities, as the protection tape tended to loosen. However, this was not considered a major problem, and overall the device together with the protection tape was considered much more hygienic than frequently taking blood samplings. Otherwise, there was no discomfort described.

“My son is an active rugby player and even though we try to attach it with extra measures it often gets removed during exercise or games”.

(R6)

“I was quite worried that she may have some allergic reaction to the tape or sensor, but she has had zero discomforts so far. She has a history with allergies”.

(R3)

Showering and bathing were not considering as causing any problems, but when swimming, the device could need extra adhesive material. Some parents had experienced malfunction episodes with the device, but nothing that seriously jeopardized the medical treatment. In these cases, the manufacturer replaced the malfunctioning device free of charge.

“I had a case when four sensors were damaged, and the manufacturer replaced all four of them”.

(R8)

Some of the parents described initial difficulties pairing the FGM device with a mobile phone, but these difficulties were perceived as primary inexperience on the part of the parents.

##### Economic Aspects

Economic concerns regarding the device were a central matter and an often-occurring theme. The Libre device is not marketed in Georgia, but those parents who had relatives or friends living in another country could buy the device abroad. The device is perceived as expensive, and not subsidized by any health care system in Georgia. Many families had to make sacrifices to be able to afford it, but the advantages expressed were so great that parents prioritized buying the device over other things.

“For Georgians, it is quite expensive and not many families can afford it. I am absolutely sure that, at least at first, and especially for smaller children, it is essential to use the Libre. It is also becoming difficult to purchase the Libre; our friend buys it for us in Germany and sends it by mail or if someone is travelling to Georgia they bring it for us. We also try to have a supply at home”.

(R1)

## 4. Discussion

The purpose of this study was to elucidate the parents’ experiences of their daily lives when a child in their family uses an FGM. In the present study, the parents experience a great improvement in daily life when they were given the opportunity to use the FGM device. Perez et al. [15] had earlier concluded that parents to a child with T1DM considered that social activities were often limited, but our informants described that, when using the FGM, children could participate in social and physical activities without complications. Similar results have shown that parents emphasized that they had more ‘normal’ children rather than those defined with disabilities in the form of diabetes [16]. This divagating result showing that, in general, children with T1DM were found to be less physically active than healthy children [17] could be due to parent’s fears of hypoglycaemia or not having the ability to change the management of diabetes and adapt this to physical activities. Exercise causes increased sensitivity to insulin and is a very important part of treatment for all individuals who have diabetes. It has been shown that increased insulin sensitivity persists for at least 8–10 h, sometimes up to 18 h or even longer after exercise [18]. Therefore, the reason why children using the FGM device were found to be more physically active, and why the parents experience an improvement in daily life, is probably due to an increased ability to manage varying blood glucose levels in a better way. 

Another expressed benefit of using FGM was in the family’s contact with the health care system, according to the history of glycaemic variability, in order to minimize later complications. Of particular interest is the ability to follow the average glycaemic variability (HbA1c) since this test shows how the blood sugar level has been during the last two to three months. The parents were convinced that the FGM helped them in their contact with a responsible physician. This is in line with Leelarathna & Wilmot [6] whose research suggests that FGM devices lead to improved pattern recognition, which is important information for the responsible physician for individualization of insulin dose adjustments. Further, a review of the challenges for parents of children with T1DM found them routinely monitoring their child’s blood glucose level at night when their child was asleep. This has an impact on their night sleep quality which in turn can lead to anxiety and deteriorating quality of life [17]. It is therefore of great satisfaction that our result describes that the FGM had improved night sleep quality for both parents and children. The advantage of not having to wake up the child during night-time for traditional measurement was considered exceptionally valuable.

It is known that parents of children with T1DM often raise complaints about self-care support during school time [19]. T1DM may impact the children’s school performance due to the demands of daily disease management in order to keep glucose levels within the target area. Present informants in our study expressed that using the FGM device gave the parents an overall sense of increased security. The parents mentioned that even the school staff felt more comfortable if the child had the device and some parents mentioned that it was a prerequisite for them to consider sending their child to day-care. A study has shown that parents often underestimate breakfast insulin so that the child does not suffer from hypo-glycemia at school [19]. However, if a situation of hypo-glycemia does occur, it must be clear to the school staff involved as to what to do and who to contact if the child needs help. If the child is older and is responsible for self-care, there must be someone who can be contacted if the condition worsens and if the child needs further help. Though it sometimes can be difficult for children with diabetes to get adequate supervision in school and/or preschool, it seems valuable that the FGM device gave an overall sense of increased security. Previous research suggests that increased training for teachers having current responsibility for children with diabetes was thought to lessen anxiety for the teachers. Greater and more accessible knowledge about diabetes for all staff was requested, and a range of practical management strategies were highlighted, including good communication and teamwork between child, parent, and school staff [20]. We, therefore, believe that the use of the FGM helps, to a great extent, to facilitate schoolwork and to reduce anxiety for parents, children, and school staff.

As the parents in the present study only had an average of seven months’ experience of the FGM, it must not be forgotten that some parents noticed skin problems in connection with FGM and CGM. However, as we interpret it, this was not considered a major problem. Nevertheless, skin problems have been highlighted in previous studies and suggests that parents should be informed they could develop and that parents and healthcare professionals should be observant and test for appropriate attachment material [21,22]. Skin irritations are probably an individual phenomenon that can/should be solved with different individual solutions. After all, skin irritations should not be neglected as they can lead to a serious infection, and insulin requirements usually increase in connection with febrile illnesses and other serious acute illnesses [23].

A significant challenge was that the Georgian parents did not have the opportunity to buy the FGM in Georgia but had to turn to relatives and friends abroad. In addition, they had to bear the costs themselves, which constituted a financial burden. This situation is not unknown, as a previous study showed that even American parents from the middle to upper-middle class experienced financial uncertainty when increased costs in connection with their children’s diabetes occurred [15]. It is important to point out that suboptimal glucose control can lead to disease complications later in life but there is also an increased risk of thyroid disorders, non-infectious enteritis and colitis, cardiovascular disease, mental illness, epilepsy, and pulmonary disease, even in childhood [24]. Therefore, it is important to make decision-makers aware of this for, as has been described, preventive work and elimination of T1DM-related complications involve significantly lower costs for the overall society [25].

### Methodical Considerations 

Obviously, the small sample size in terms of the number of informants is a limitation, and fewer male than female parents were included in this study. However, in general, the answers of the male and female parents were similar. Further, in our study, purposive sampling was used with the disadvantage that the result is more difficult to generalize. However, all informants that responded to the questionnaire had the opportunity to clarify their positions in follow-up dialogues. We believe that the answers to the questionnaire were genuine, and of importance to the informants, which we believe strengthens our results. 

Another limitation could be our translation process, though the data were translated from the Georgian language to English. The described translation process was carried out by a Georgian native with one year of scholarship in an English-speaking country. We therefore believe that the methodical process of analysis increased the credibility of the interpretation study through the fact that the coherency of interpretations was discussed and revised by all authors. Still, since the interpretation of qualitative results has a subjective component, it is up to the reader to evaluate the transferability of the results.

## 5. Conclusions

The results of the present study show that parents of children with TIDM experienced a great improvement in quality of daily life when they were given the opportunity to use the Libre device. Everyday life changed positively as the children’s blood glucose levels were perceived to be better adapted to their illness and the parents experienced better opportunities for their children within the school and other social activities. Implementing the Libre device in diabetic care for Georgian children could be a major step toward the European medical quality standards.

## Figures and Tables

**Table 1:** Demographic parents (informants) and children characteristics, in absolute (*n*) and relative frequencies (%). Length of diabetes disease, and experiences of the Libre device in month (medians and interquartile range (IQR)).

Parents N = 20		Children N = 20		Length of Diabetes Disease & Libre Experience (Month)
**(** ***n*** **/%)**		**(*n*/%)**		**Length of diabetes disease**
Men	3 (15)	Boys	9 (45)	(median (IQR)) 17 (6–62.25)
Women	17 (85)	Girls	11 (55)	(median-year & month) 1 year 5 month
				min-max 4–96
**Age (year)**		**Age (year, (*n*/%))**		**Libre experience**
mean ± SD	38 ± 4	0–3	2 (10)	(median (IQR)) 7 (3.25–30)
min–max	30–45	4–7	7 (35)	min–max 1–72
		8–12	7 (35)	
		12–16	4 (20)	

N (=total population) because it is all parents. A mall *n* represent a part of a population.

## Data Availability

The datasets generated and/or analysed during the current study are not publicly available to protect the personal integrity of the patients but are available in redacted form from the corresponding author on reasonable request.

## Appendix A. Included Questions in Used Questionnaire Survey

1. What does the Libre device mean to your child and your family?
2. What feature of the Libre device is most useful for you and your child?
3. How do you perceive the device importance for your child at school/kindergarten?
4. Have your child’s medical parameters (mostly HbA1c) improved or changed in any way?
5. Do you feel safer when your child uses the Libre device (hyper-/hypo-glycaemia)?
6. How much do you think the use of Libre device helps you/your child with decision making for proper insulin dosing? Eating? Physical activity?
7. What is your experience according to the glucose control when stress situations and acute illnesses occur (using the Libre device)?
8. How do you think the use of the Libre device effects your child’s coping to the disease?
9. How do you think the device has affected/facilitate your child’s hobbies and favorite activities?
10. What are your thoughts about the otherwise society’s perception (in general), when your child are using the Libre device?
11. In what way affect the Libre device personal hygiene and self-care?
12. If you have had device malfunction episodes, could you please describe them?
13. Have you found the alarms for high/low useful?
14. What features do you think are lacking?
15. When I say costs for the Libre device, what comes to your mind?
16. As a parent, estimate your *total satisfaction* with the Libre device using a Likert scale 1–10 (1 = not at all satisfied-10 = totally satisfied).
